# “I literally had no support”: barriers and facilitators to supporting the psychosocial wellbeing of young people with mental illness in Tasmania, Australia

**DOI:** 10.1186/s13034-023-00621-y

**Published:** 2023-06-09

**Authors:** Melissa Savaglio, Marie B. H. Yap, Toni Smith, Ash Vincent, Helen Skouteris

**Affiliations:** 1 Health and Social Care Unit, School of Public Health and Preventive Medicine, Monash University, 553 St Kilda Road, Melbourne, VIC 3004 Australia; 2 Turner Institute for Brain and Mental Health, School of Psychological Sciences, Monash University, Melbourne, VIC Australia; 3 Melbourne School of Population and Global Health, University of Melbourne, Melbourne, VIC Australia; 4 Baptcare, Hobart, TAS Australia; 5 Warwick Business School, University of Warwick, Coventry, UK

**Keywords:** Youth mental health, Psychosocial wellbeing, Qualitative, Youth voice

## Abstract

**Background:**

There has been limited focus on understanding the barriers and facilitators to meeting the broader psychosocial needs of young people with mental illness from the perspectives of young people. This is required to advance the local evidence base and inform service design and development. The aim of this qualitative study was to explore young people’s (10–25 years) and carers’ experiences of mental health services, focusing on barriers and facilitators to services supporting young people’s psychosocial functioning.

**Methods:**

This study was conducted throughout 2022 in Tasmania, Australia. Young people with lived experience of mental illness were involved in all stages of this research. Semi-structured interviews were conducted with 32 young people aged 10–25 years with experience of mental illness, and 29 carers (*n* = 12 parent–child dyads). Qualitative analysis was guided by the Social-Ecological Framework to identify barriers and facilitators at the individual (young person/carer level), interpersonal, and service/systemic level.

**Results:**

Young people and carers identified eight barriers and six facilitators across the various levels of the Social-Ecological Framework. Barriers included, at the individual level: (1) the complexity of young people’s psychosocial needs and (2) lack of awareness/knowledge of services available; at the interpersonal level: (3) negative experiences with adults and (4) fragmented communication between services and family; and at the systemic level: (5) lack of services; (6) long waiting periods; (7) limited service accessibility; and (8) the missing middle. Facilitators included, at the individual level: (1) education for carers; at the interpersonal level: (2) positive therapeutic relationships and (3) carer advocacy/support; and at the systemic level: (4) flexible or responsive services, (5) services that address the psychosocial factors; and (6) safe service environments.

**Conclusions:**

This study identified key barriers and facilitators to accessing and utilising mental health services that may inform service design, development, policy and practice. To enhance their psychosocial functioning, young people and carers want lived-experience workers to provide practical wrap-around support, and mental health services that integrate health and social care, and are flexible, responsive and safe. These findings will inform the co-design of a community-based psychosocial service to support young people experiencing severe mental illness.

## Introduction

Youth mental illness remains a global public health challenge. In Australia, mental illness is one of the leading causes of burden of disease among youth (young people, aged under 25 years), with suicide the leading cause of death among 15–24-year-olds [1]. In Tasmania, Australia, the rate of youth mental illness is significantly higher than the national average [1, 2]. Most recent estimates indicate that the percentage of young people experiencing a mental illness in Tasmania has significantly increased from 10.6% in 2012 to 18.8% in 2018 [3]. Tasmania’s rate of youth suicide is also higher than the national average [4]. These rates of mental illness exist within the context of a state whose youth experience significant vulnerability, compared to the rest of Australia. Tasmania has the most rurally and remotely dispersed population of youth in Australia [5]. The Index of Relative Social Disadvantage indicates that Tasmania has the highest proportion of youth living in the most socio-economically disadvantaged areas of the country [3]. Approximately 68% of young people in Tasmania live in local government areas of greatest disadvantage, characterised by low income, low educational attainment and high unemployment [3]. Rates of youth homelessness are also higher than the national average [3]. Despite significant need, Tasmania has one of the most underdeveloped and under-resourced youth mental health sectors in Australia [6]. Recent service mapping of community-based youth mental health services in Tasmania identified limited availability, accessibility, and capacity of existing services to appropriately and holistically support youth experiencing mental illness [7]. Nonetheless, local reform is underway to better support young people’s psychosocial wellbeing. Exploring the perspectives and experiences of young people and carers is needed to inform the (re)development and (re)design of mental health services in the state.

Despite high prevalence of youth mental illness, service utilisation rates remain low. The gap between the need and access to mental health support is larger for youth than any other age group [8], with approximately 70% of young people not engaged in any services nor receiving the support that they need [9]. This lack of mental health service use by young people is concerning, as it can lead to exacerbated mental health concerns and psychosocial disability that persist into adulthood [10]. Low service uptake among this cohort has been attributed to a range of help-seeking experiences and barriers to accessing and engaging in services [8, 11]. Such barriers include limited service accessibility and availability (due to high out-of-pocket costs and long waiting times), lack of services, difficulty navigating the mental health system, stigma/embarrassment around help-seeking, negative past experiences with services (i.e., dismissive experiences with professionals, not having needs met, inconsistent care etc.), and lack of awareness of mental health concerns or services [8, 11–15]. However, Australia is underrepresented in reviewed literature to date [11, 13]. Identifying the barriers and involving young people in designing potential solutions to ameliorate such barriers is crucial to ensuring more young people can receive timely and appropriate supports and services that they need to enhance their trajectory.

Psychosocial wellbeing refers to young people’s broader functioning, such as social functioning, education, community engagement and day-to-day living. Nearly a quarter of Australian young people who experience mental illness report the psychosocial impact of their mental illness as ‘severe’ [1]. A decline in functioning often triggers young people or caregivers to seek mental health services, whilst improvement in functioning can indicate the effectiveness of a mental health intervention [16]. Sustained mental illness and poor psychosocial functioning can impact young people’s life trajectory into adulthood, and are associated with adverse outcomes if not addressed, such as unemployment, socioeconomic stress, social and community isolation, and criminal justice involvement [16]. Therefore, a holistic approach to youth mental health intervention and models of care that consider structural factors, relationships, and individual needs in their design and delivery, is crucial to address the various underpinning factors and broader functional impact of mental illness experienced by young people. Specifically, Bronfenbrenner’s [17] Social-Ecological Framework has been used to explain how children’s development are shaped by their interactions with others and their surrounding context, including the people, environment, communities, and systems in which they live [17]. This framework can help explain the complex interplay between the various levels of factors that influence young people’s psychosocial wellbeing, and to guide recommendations to ensure that new solutions or interventions address existing barriers and enhance facilitators across multiple levels [18]. However, there has been limited focus on adopting a social-ecological approach to exploring and meeting the psychosocial needs of young people experiencing mental illness to date.

Whilst patient and public involvement is an essential part of public health research, meaningful involvement of young people with mental illness in research is lacking [19, 20]. Supporting young people to be involved in all stages of the research process is crucial to empowering them to reshape and redesign youth mental health services, and ultimately enhance outcomes. It is also necessary to include carer voices, because for many young people, their carers are the main conduits for accessing mental health support [13, 21]. Understanding both carer and young peoples’ experiences and triangulating their perspectives, as key consumers of the youth mental health system, is crucial to informing holistic care. Further, to the authors’ knowledge, there has been limited recent research exploring young people’s experiences of mental health services in Australia, particularly in Tasmania. There has also been limited focus on understanding the barriers and facilitators to meeting the broader psychosocial needs of young people with mental illness, from the perspectives of young people and carers combined. This knowledge is required to advance the local evidence-base and inform service design and development in Tasmania, to ensure the unique needs of the local community are met. Therefore, the aim of the current study was to explore young people’s (10–25 years living in Tasmania) and carers’ experiences of mental health services, focusing on the barriers and facilitators to supporting their psychosocial functioning.

## Methods

### Study design

This qualitative study was approved by the Monash University Human Research Ethics Committee, and reporting was guided by the Consolidated Criteria for Reporting Qualitative Research [22]. This study forms part of a comprehensive needs assessment for a larger project co-designing a psychosocial service for young people experiencing mental illness. An advisory group of six young people aged 18–25 years with experience of mental illness and service involvement in Tasmania were involved throughout each stage of this research, including informing the study’s aim and research questions, defining participant eligibility criteria, choosing recruitment and data collection methods, developing recruitment materials, and refining the key themes. Young people ensured that ample flexibility and choice were provided to engage the target sample in this study. One of these young people also co-authored the current paper.

### Participants

Purposive sampling was used in this study, where young people aged 10–25 years with a living experience of mental illness and engagement with services in Tasmania were eligible to participate. Carers of a young person who met the criteria were also eligible to participate. A multi-pronged approach to recruitment was employed, via existing mental health consumer engagement organisations and peak bodies, which provide advocacy, representation, and information for people experiencing mental illness (e.g., Flourish, Mental Health Council), youth mental health services across the state (e.g., Pulse, the Link, CAMHS), and general youth community networks (e.g., Youth Network of Tasmania, local council groups). Recruitment was conducted via multiple modalities, including various social media platforms of local youth services and organisations, in-person flyers on-site at various existing services, and via emailing lists of consumer engagement groups, which targeted carers (e.g., Mental Health Family and Friends). A total of 65 individuals expressed interest to participate, of which four did not proceed to interview due to change of mind (*n* = 1) or did not respond to researcher attempts to organise an interview (*n* = 3). The final sample comprised of 61 participants, including 32 young people and 29 carers, of which 12 were carer-child dyads. Parental consent was sought for children under 16 years.

Tables 1 and 2 describe the characteristics of young people and carers who participated in this study. Young people were aged 11–24 years, 17 years on average, were predominantly female and lived in South Tasmania. Carers were 52 years on average, between 39 and 64 years, and were predominantly the mother of a young person with mental health concerns. There was a high level (82%) of mental health comorbidity among young people. The most common concerns were anxiety (88%), depression (76%), and psychosis (35%). Young people also noted various psychosocial needs, reflecting the complexity of their presentation, including social isolation or disengagement, complex family relationships, disengagement from education or difficulty completing schoolwork, physical inactivity, and housing/financial stressors. The majority (73%) were engaged with supports at the time of interview, yet all had recent experiences (last 12 months) of attempting to access mental health services in Tasmania.

**Table 1:** Participant demographic summary

	Young people (*n* = 32)	Carers (n = 29)
Age		
Mean age (SD)	17.4 (2.3)	52.9 (3.7)
Age range	11–24	39–64
	N (%)	N (%)
Gender		
Female	21 (66)	22 (76)
Male	9 (25)	5 (17)
Non-binary	2 (6)	0
Location		
South	14 (44)	15 (52)
North	11 (34)	8 (28)
North West	7 (22)	6 (21)
Aboriginal	6 (19)	4 (13)
LGBTQIA+	5 (16)	0
Relationship to young person	NA	
Mother		23 (79)
Father		5 (17)
Grandmother		1 (3)

**Table 2:** Summary characteristics of young people

Characteristics	Young people (n = 49)^a^ N (%)
Mental health concerns^b^	
Anxiety	43 (88)
Depression	37 (76)
Early psychosis	17 (35)
ADHD	6 (12)
OCD	5 (10)
PTSD	3 (6)
Personality disorder	3 (6)
Eating disorder	2 (4)
Comorbidity	40 (82)
Psychosocial needs^b^	
Social isolation/disengagement	28 (57)
Complex family relationships/dynamic	25 (51)
School/uni disengagement	20 (41)
Physical inactivity	19 (39)
Physical/chronic health concerns	16 (33)
Homelessness/unstable housing	14 (29)
Drug and alcohol use concerns	14 (29)
Financial stressors	10 (20)
Unemployment (over 18)	8 (16)
Poor nutrition	5 (10)
Child protection involvement	1 (2)
Youth justice involvement	1 (2)
Currently engaged in support	36 (73)

^a^*N* = 49 as *n* = 12 young people were part of a dyad (both the young person and their carer participated) so their characteristics are only included once

^b^Majority of participants reported more than one mental health concern and psychosocial concern so % does not equal 100

### Data collection

Participants had the choice to participate in a semi-structured one-on-one or dyad interview (carer and child together) with the researcher, or a focus group with other young people or carers, depending on their personal preference. Participants chose the time, place and modality of the interview, including in-person, via phone, or over Zoom. All participants engaged in a one-on-one interview with the researcher, except two carer-child dyads who each participated in a dyad interview. Interviews were generally conducted during after-school/work hours or weekends, either via phone or Zoom. Young people and carers were asked about their experiences of accessing and engaging in mental health services, the barriers and facilitators that helped or hindered their utilisation of mental health services, the extent to which such services met their psychosocial needs, suggestions for how to overcome their identified barriers and recommendations for how services can better facilitate their psychosocial wellbeing. Interviews were semi-structured to allow for prompting and tailoring of questions based on each participant’s unique experience. Participants were each given a $30 voucher for their time and involvement. Interviews were approximately one hour in duration, all conducted by a female researcher, and were audio-recorded. Interviews were transcribed verbatim by the researcher and provided to participants for feedback/correction.

### Data analysis

Deductive content analysis was conducted to identify the various barriers and facilitators to supporting young people’s psychosocial wellbeing. This analytic approach was chosen to effectively categorise the content discussed by participants as a barrier or facilitator, based on existing knowledge [23]. Bronfenbrenner’s Social-Ecological Framework [17] was chosen at the data analysis stage to be used as a guide to group barriers and facilitators across the various levels within which the young person exists and interacts with—individual (factors relating to the young person or the carer themselves), interpersonal (relationships with clinicians, family, peers) and systemic level (factors related to mental health services, sector, structures). This framework was used to help explain the complex interplay between the various levels of factors that influence young people’s psychosocial wellbeing, and to guide recommendations to ensure that new solutions or interventions address the various identified barriers and enhance facilitators across multiple levels. Data collection and data analysis were conducted concurrently to assess for data saturation in real-time. Saturation was reached after the 49^th^ interview, yet the researcher continued interviews with the 12 remaining participants who had initially expressed interest in participating. Transcripts were coded by the same researcher who completed the interviews, and 50% were double-coded. Each coder independently coded and developed their own codebook, then the two researchers engaged in a collaborative discussion to compare codes, ensure consistency, and to group codes into various themes. The initial themes were then shared with the advisory group of young people, who engaged in an iterative process of collaboratively developing, refining and naming the themes.

## Results

**Fig. 1:**
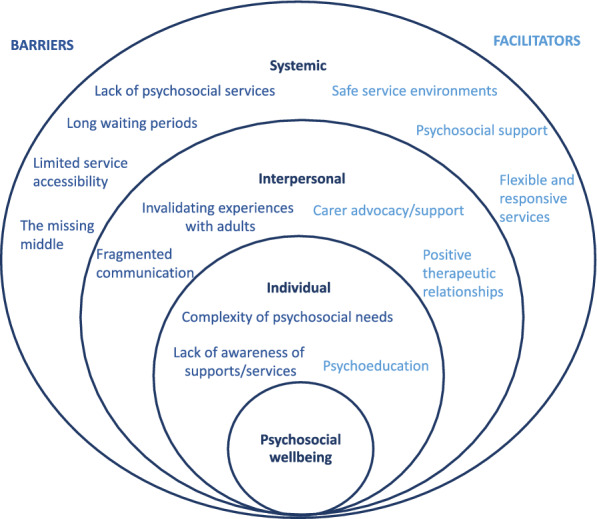
Barriers and facilitators to services supporting young people’s psychosocial wellbeing across the social-ecological model

A total of 14 subthemes were identified, including eight barriers and six facilitators across the individual, interpersonal, and systemic levels. Quotes are included from young people (YP) and carers (C), with the age of the young person identified (‘C, 14yo, male = carer of a 14-year-old boy). Figure 1 presents a visual summary of barriers and facilitators across the various levels of the Social-Ecological Model.

### Barriers

#### Individual young person/carer level

1. Young people’s psychosocial needs: “there’s so much going on”

Carers identified that the psychosocial functional impact of the young person’s mental illness was a barrier in itself to accessing support. The complexity, severity, and impact of mental illness influenced young peoples’ capacity, readiness, and motivation to access and/or meaningfully engage in support.

- *He’s not motivated* [to engage] *at all. I can’t even get him out of bed, let alone to a service.* C, 14yo, male

For young people, this was described as a sense of feeling overwhelmed or overburdened by the functional impact of their mental illness combined with the pressures of everyday living and the thought of accessing and engaging in services.

- *How can you even focus on getting help when you're worried about things like your housing, generally being able to eat throughout the week, because you can't afford it, or getting a job? There’s so much else to think about when you've got these kinds of stressors. Accessing support is a really big task to undertake when you're mentally ill and struggling to function.* YP, 20yo, female

- *A lot of us are really struggling down here, and we have been for a while. Like there’s no housing, and now cost of living is insane…We’re genuinely worried about getting our basic needs met like food, money, and a roof, so getting support for mental health isn’t always at the top of the list.* YP, 23yo female

2. Lack of awareness of supports: “I didn’t know where to go”

Young people and carers reported that a lack of awareness of available and appropriate services and not knowing how or where to access support, often led to delayed help-seeking and increased severity of concerns.

- *I honestly had no idea what I was doing. It was like going down a rabbit hole of confusion.* YP, 15yo, female
- *If we had known where to go from the start, maybe she wouldn’t have been hospitalised.* C, 13yo, female

Some carers reflected that low mental health literacy among caregivers and the local community more broadly across the state may partly contribute to this lack of awareness.

- *“I work in the sector and it was still incredibly hard for me to get her in, so what about the majority of the parents across Tassie who would have no idea what to do or where to go? It’s really difficult.”* C, 14yo, female

Carers also discussed the burden of accessing supports for their child, and how multiple unsuccessful attempts of help-seeking had an adverse impact on their own wellbeing:

- *The lack of available options for my child has affected my mental health as well. *C, 17yo, male
- *We were at a loss about how do we get support for him? Everywhere we went they said, ‘nope can't help you’, so I was pretty broken down by that stage…It’s taken a massive toll. I'm in grief. I hurt inside. *C, 15yo, male

#### Interpersonal level

1. Invalidating experiences with adults: “no one would believe me”

Young people frequently recounted negative experiences of their concerns being dismissed, invalidated, or misunderstood by adults, including their carers and clinicians.

- *My parents basically thought that my mental health issues weren’t a real thing, they just didn’t believe me and didn’t get how serious it actually was until I ended up in ED.* YP, 19yo, female
- *I’ve seen probably over half a dozen different people, and very rarely were they actually helpful. Most of them I didn’t really feel like I could talk to them at all, and they just didn’t get how serious I was.* YP, 17yo, female

Some carers also had negative appraisals of their child’s clinicians, including not listening to their young person’s concerns, providing inconsistent care, and being unsupportive.

- *He basically said that our daughter was never going to get better, she was likely to end up on the street, end up on drugs, she was likely to fall in with people who would rob us. That was just a really horrible experience.* C, 18yo, female

Such experiences prevented or delayed young people from accessing support, resulting in elevation of mental illness (i.e., crisis), further decline in psychosocial functioning, and/or discontinuing their service engagement.

- *I was like, “no mum, like you don't understand, this is making me not want to be alive”. She was always like, “you’re just being dramatic”. I guess she didn't understand or almost didn't believe it in a way. She didn't start to listen until after my suicide attempt in grade nine.* YP, 20yo, female
- *I didn't think they understood how serious it was because they kind of just put my name down for a session and then turned me away, even though I was at my lowest point. I never went back there.* YP, 22yo, female

2. Fragmented communication: “there’s lots of mixed messages”

Fragmented and inconsistent communication from mental health services was a predominant challenge faced by carers. Specifically, carers reported considerable difficulty with communication and confidentiality once their child turned 18.

- *Last week I was able to talk to them about how I'm scared he's gonna hurt himself. Then next week he’s 18, I still think he's gonna hurt himself, but I get ‘sorry, can't talk to you.* C, 18yo, male
- *I understand the need to respect the confidentiality, but our daughter was very upfront about you can tell my parents. She really would have benefited from keeping us more in the loop of what was going on, but they didn't do that at all.* C, 15yo, female

Young people valued the confidentiality and privacy practices upheld by services, while some had difficult experiences when family became involved without their consent.

- *It was nice to have someone that I could trust and wouldn't go tell my parents, ‘oh he's been smoking weed’ and stuff like that.* YP, 16yo, male
- *Home was never really a safe place for me so once they told mum, I ended up having to move out of home a few days after turning 17. It just made everything worse and I didn’t go back.* YP, 21yo, female

Carers wanted services to maintain transparent and consistent communication with families, where appropriate and as desired by the young person.

- *I think it would have been really good for our daughters to have had the option for us to have been more involved in their care. They are both at home and we have provided a very stable, resilient, supportive home for them. I know that's not always the case for kids with mental health issues. But for our situation, it would have been really helpful for them to keep us in the loop more.* C, 17yo and 19yo, females

#### Systemic level

1. Lack of services: “It’s just the tip of the iceberg”

Young people and carers acknowledged the lack of holistic psychosocial supports/services in Tasmania, which was identified as a major gap to supporting young people’s broader functioning and recovery from mental illness.

- *That broader psychosocial support is missing in the youth mental health space, like no one’s helping set them up for recovery and their future.* C, 17yo, female
- *Tassie has nowhere near the amount of services that we actually need. In the North-West, there's basically nothing for us.* YP, 18yo, male
- *If your child doesn’t qualify for NDIS* (National Disability Insurance Scheme, provides psychosocial support to people with a disability*), it's nearly impossible to get that more intensive support…he needs help with education, healthy eating, maybe job opportunities, budgeting, getting his licence…but there’s no one in Tas to help with that.* C, 16yo, male

Young people and carers also identified the lack of clinicians in Tasmania as a key barrier to accessing any support. Participants recognised that the mental health workforce shortage in Tasmania is a significant barrier, with many services relying heavily on locum specialists from the mainland.

- *I think one of the biggest challenges in Tasmania is we don't actually have enough workers, so if you can't find someone to click with, you’re screwed because there’s not many other options.* YP, 22yo, female
- *Psychologists are great, but there's not many of them here and only so much they can do. You’re lucky to get monthly, so what’s happening in the in-between? More holistic support is needed.* C, 14yo, female
- *The pool of specialists here is very small. She was engaged with* [Tasmanian service] *but they could only do telehealth because the psych was based in Victoria—there was no one in-person that she could see here.* C, 13yo, female

Both young people and carers recognised that the currently available services were only addressing ‘the tip of the iceberg’, as they did not have capacity to provide in-depth, long-term, consistent holistic support that they felt was required.

2. Long waiting periods: “There’s so much waiting”

All young people and carers identified that long waiting lists for services, which have been exacerbated since the COVID-19 pandemic, have prevented or delayed engagement with supports.

- *You can't be told that your very unwell and still suicidal child has to wait 2 months to see someone after leaving hospital. What are we supposed to do in the meantime?* C, 16yo, male
- *Just getting into services is a headache. Even if you find the ‘right’ service, you have to wait months and months to get seen. You make that decision when you're quite low, and you really need it then and there*. YP, 23yo, female

Participants recounted numerous experiences where long waiting times (i.e., up to 9 months) had contributed to mental health escalation/crises, functional decline, increased severity, and being no longer eligible for the intended service.

- *She deteriorated as she waited and ended up having a suicide attempt…when they finally contacted her they said, ‘oh you’re too high risk now, we can’t take you’. Then she’s back to square one with no support but she’s more severe. It’s ridiculous.* C, 15yo, female
- *While you’re waiting, your mental health is worsening…I actually ended up in hospital three times after that initial time, because there just wasn't any care.* YP, 18yo, male

3. Limited service accessibility: “accessing support is a nightmare”

Young people consistently reported difficulties accessing services due to the location, distance and amount of travel required to attend.

- *I had no one who could take me to appointments, and I live about an hour and 40 min by bus from Hobart CBD, so I had to stop because it was just too far away.* YP, 17yo, female
- *I wasn’t able to attend in-person, the closest service to me is over an hour away, it’s even longer by bus. And I know there’s worse-off kids than me further North who can’t get any help.* YP, 20yo, male

Participants also identified further logistical barriers to accessing supports, including costs and lack of after-hours support:

- *When I was in high school, my parents were happy to pay for my psychologist. But then when I went to uni and I was living out of home, it would have been good to see someone but it wasn’t even an option because of the cost.* YP, 24yo, female
- *We’re fortunate to be able to afford private mental health services, but this would be well out of reach for most families.* C, 11yo, female
- *There are no psych clinics here that bulk bill.* YP, 19yo, female
- *We've been sitting on the edge of our seat thinking what do we do after hours? God forbid he has a relapse psychotic episode after five or on a weekend.* C, 16yo, male

These logistical factors significantly limit the availability and accessibility of mental health services for young people in Tasmania.

4. The missing middle: “we’re stuck in the middle”

The majority of participants experienced significant difficulty accessing an appropriate service. Termed the “missing middle” by participants, young people consistently felt that they were being “bounced around services” as a result of their mental health severity not meeting various service eligibility criteria.

- *I was told I’m too high risk for headspace but not risky enough for CAMHS, but there was nowhere else that would take me, so I literally had no support.* YP, 16yo, female
- *I found that there’s lots of places that will help if you're in kind of crisis or need low-level therapy support, but there isn’t just that middle spot where you need that ongoing holistic support.* YP, 20yo, female
- *He’s had constant rejection by community mental health services from aged 14. Then when he finally met their threshold at 17, they rejected him again as he was smoking dope.* C, 19yo, male

The lack of services for the ‘missing middle’ has resulted in young people and carers navigating a complex and fragmented system with limited success.

- *Even when they say no, sometimes they wouldn't even do a referral to a new place so you would have to sort it out yourself…when your mental health is that awful, and you’re homeless, and you’re 17 years old, that is just not good enough.* YP, 19yo, male
- *Continuously being told by services that they can’t help you is so frustrating, especially if you're currently experiencing quite severe mental illness. It's difficult enough to navigate for someone who's in a good head space.* YP, 21yo, female
- *“Community mental health here in Tasmania just doesn't really have a pathway. They can’t do stepping stones and referrals across services because the appropriate services for young people don’t even exist.* C, 12yo, male

### Facilitators

#### Individual young person/carer level

1. Education for carers: “support the supporter”

Carers requested more information, awareness, or psychoeducation from services to help them meet their children’s psychosocial needs at home, particularly if they were experiencing challenges engaging with services.

- *We are informed and resilient, but many people are not. Most carers need practical support with managing and understanding the situation to help their young person and support for their own resultant mental health issues.* C, 19yo, female
- *It would be good to have information on how to refer, knowing the who and how of making contact and what kind of support is available, and education and support around caring for/reaching out to your child.* C, 12yo, female
- *Seeing a psychologist one hour in a week is not enough. There's got to be stuff happening in between at home, so as a parent, it would be helpful to know what can I do that might help.* C, 14yo, male

Young people did not discuss this topic, yet carers consistently felt they needed more support to improve their mental health literacy and help them care for their child at home.

#### Interpersonal level

1. Positive therapeutic relationships: “find someone you click with”

Young people and carers emphasised the importance of a positive, validating, trusting, and consistent relationship with their clinician/support person.

- *She’s my counsellor but she’s also like my friend now, so I feel safe and comfortable talking to her about anything.* YP, 17yo, female
- *It’s the best when it feels like you’re just like talking to a friend and getting a coffee, especially once it’s someone regular and I don’t have to retell my whole story every time.* YP, 22yo, male

Young people described feeling safe, comfortable, and motivated to engage when they were ‘matched’ with someone with a similar ‘vibe’, who was respectful, supportive, non-judgemental, and friendly.

- *We were just a good match, like similar personality, and I felt like she really got me.* YP, 16yo, female
- *Knowing that they're also a part of the LGBT community makes it just feel a lot more comfortable and safe, because they’ve had some of the same experiences as me, and I know that they understand me and they’re not judging me.* YP, 22yo, non-binary

Participants also consistently recommended that services include peer support workers with lived experience of mental illness to better engage and address the broader psychosocial needs of young people experiencing mental illness. Young people felt that diverse lived-experience workers could more effectively provide that validation, acceptance, positive representation and unconditional support that they desire.

- *It would be good to have a mentor who’s gone through something similar and come out the other side. They would be super compassionate, understanding, accepting and validating.* YP, 18yo, female
- *We need youth peer workers here. They'll be able to work more flexibly and they'll probably have more success on the psychosocial side of things, like helping kids resume some activities or play a game of basketball or go shopping with them, get them out of the house.* C, 16yo, male

2. Carer advocacy and support: “it’s a case management role”

Both young people and carers recognised that having a family member to support, persist, and advocate against various systemic obstacles was instrumental to accessing support.

- *I have essentially been case managing my daughter and I didn’t give up. Without that, she'd probably be dead.* C, 17yo, female
- *I know that I’m very lucky to have very understanding and supportive parents. I definitely wouldn’t have come this far without them.* YP, 21yo, female

Young people who did not have any family support valued having a support person/worker to provide more practical holistic support and advocacy on their behalf.

- *Honestly having someone in my corner would have made the world of difference. Instead of being alone, someone who was there for me, I could ask questions, someone to count on, to talk to and help me out.* YP, 19yo, female

#### Systemic level

1. Flexible and responsive services: “support that meets our needs”

Young people and carers agreed that services need to be flexible and responsive, in terms of how support is accessed and delivered. Participants emphasised the value of services providing various accessibility and engagement options, including outreach, drop-in, telehealth, and informal activities.

- *More flexible access, because I didn’t want to let my parents know, so it was really hard to go to a physical place regularly. I found it easier when things were online and also having a drop in space, because I didn’t have to make an appointment.* YP, 18yo, male
- *More services need to deliver outreach, especially for us here and others in the rural areas, which is probably a large chunk of the state, they really need to get out in the community more.* C, 13yo, female
- *It would be awesome if there was a person that physically came and checked in on her, or went for a walk or engaged her in some activities, like music or sport.* C, 15yo, female

Young people also wanted support that better met their needs, in terms of the frequency, intensity, timing, and duration of support, including the potential for service provision to cover after-hours/weekends and longer duration of support over 12 months.

- *She was literally always available…it was so comforting to know that I didn’t have to wait weeks and weeks in-between sessions…the regular contact was really good for me.* YP, 23yo, female
- *I think more long-term and consistent support is needed to actually make a difference. It would be ideal if they could spend a couple hours with her each week, rather than in and out.* C, 16yo, female

Ultimately, young people valued services that gave them choice, and empowered them to engage when and how they needed to enhance their psychosocial wellbeing.

2. Psychosocial support: “address the root causes”

Participants recommended that services better address the contextual factors and stressors that are underpinning or impacted by young people’s mental health. Young people and carers both spoke about the need for holistic and integrated services that can support young people’s psychosocial wellbeing and provide respite for their carers.

- *It would be really great to have services that address the root causes of young people's mental illness and all the life things that just don’t get addressed. Like help us to apply for housing or find safe accommodation or apply to uni.* YP, 19yo, male

- *I think we need to have peer workers or support workers assisting with some of the things that a psychologist might recommend, like taking me out on public transport for exposure, helping out with remembering to take your medication or picking up medication, getting a job, finding a rental, cooking skills, all those things are so much harder with mental illness.* YP, 22yo, female

- *Some sort of support worker I think that would be really helpful to have someone to do all that organising and linking with other services, and just to be there whenever you need.* YP, 17yo, female

Participants envisaged this type of service to be embedded alongside or within a mental health support/team (i.e., psychiatrist, psychologist) to provide integrated health and social care support via a multidisciplinary team. Carers also recommended this type of support, and acknowledged it would provide some respite for them too.

- *I would actually benefit from knowing that he's got a support person who can just take the pressure off us for a bit, even a few hours a week would be good.* C, 16yo, male

3. Safe service environments: “feeling safe, supported, and connected”

Participants acknowledged that the environment in which support is delivered can influence the young person’s willingness to engage. Young people reflected on positive experiences attending services that gave them a sense of safety (including cultural safety), community, were non-clinical and home-like, and provided opportunities for connection with their peers.

- *Just a safe space—that’s what I look for and notice straight away.* YP, 19yo, female
- *Little things like the admin staff asking me my pronouns and making sure that other people are respecting the pronoun, it’s gender-affirming and it makes a huge difference.* YP, 24yo, non-binary
- *Safe spaces and opportunities for ongoing informal support from diverse staff in a more relaxed and pleasant setting integrated within the core mental health services.* C, 21yo, female

Young people and carers consistently advised that more youth-friendly services are needed across the Tasmanian youth mental health sector. Young people consistently reinforced that having more diverse lived-experience workers to increase representation of various identities (e.g., LGBTQIA+, Aboriginal or Torres Strait Islander, mental illness, culturally and linguistically diverse) may create more welcoming services in which young people feel safe, seen, heard, represented and respected.

## Discussion

This study explored young people’s and carers’ experiences of mental health services in Tasmania, with a focus on their perceived barriers and facilitators to supporting young people’s psychosocial wellbeing. Barriers included, at the individual young person/carer level: (1) the complexity of young people’s psychosocial needs and (2) lack of awareness of supports; at the interpersonal level: (3) invalidating experiences with adults and (4) fragmented communication between services and family; and at the systemic level: (5) lack of services; (6) long waiting periods; (7) limited service accessibility; and (8) the missing middle. Facilitators included, at the individual level: (1) education for carers; at the interpersonal level: (2) positive therapeutic relationships and (3) carer advocacy/support; and at the systemic level: (4) flexible and responsive services, (5) services that provide psychosocial support; and (6) safe service environments. The findings are similar to those reported in previous international reviews that identify numerous systemic barriers to young people getting the relevant clinical support that they require [8, 11–13]. This study extends previous findings by focusing on meeting the psychosocial needs of young people, exploring the Tasmanian mental health context, and including young people in the research.

Participants identified a dearth of services in Tasmania to appropriately support the psychosocial functioning of young people experiencing mental illness. This was also confirmed in recent service mapping of community-based mental health services across Tasmania, as existing services do not have the capacity, integration, diversity and accessibility to meet the need [7]. Young people and carers acknowledged that existing services are only addressing the ‘tip of the iceberg’, and consistently called for greater holistic support, including schooling, work, activities of daily living, engaging in community/hobbies/activities, housing, and healthy lifestyle behaviours to address the increasing psychosocial complexity, stressors, and underlying socioeconomic factors of young people in Tasmania requiring support. This is particularly relevant for young people in the ‘missing middle’ who felt that current services did not meet their clinical or psychosocial needs. Worldwide, ‘the missing middle’ poses a public mental health crisis of meeting the needs of young people who require more intensive holistic support, with nearly 75% of young people with severe mental illness who should qualify for entry to community-based mental health services being denied [24]. This cohort requires more holistic, long-term, flexible, integrated support to address the psychosocial underpinnings and impacts associated with mental illness. Internationally, there are few but key examples of services aiming to support the broader psychosocial needs of young people experiencing severe mental illness, termed integrated community-based youth service hubs or ‘one-stop-shops’ [25, 26]. These include New Zealand’s Youth One Stop Shops [27], Youth Wellness Hubs in Canada [28], and Jigsaw in Ireland [29], which provide clinical mental health support and social care services embedded in a single community-based setting delivered by a multidisciplinary team, aiming to ameliorate care fragmentation, service navigation and accessibility barriers for youth. However, there is a lack of such models that have been implemented and evaluated within Australia [30]; Orygen Youth Health in Victoria recently launched psychosocial support packages for young people aged 16–24 years experiencing moderate to severe mental illness [31]. It is clear that greater integration within the Tasmanian youth mental health sector is required so that young people have access to both clinical and social care support to enhance their psychosocial wellbeing.

Young people and carers identified numerous service accessibility barriers, including long waiting lists, costs, and limited opening hours. Such barriers have been well-documented [12, 32], and they have become even more pertinent since COVID-19, with youth mental health services worldwide under significant strain to meet the increasing demand [33]. Nonetheless, the most predominant accessibility barrier identified in this study was the difficulty travelling to appointments (i.e., due to distance, no carer to take them). Tasmania has a highly dispersed population, and large proportion of youth living in rural and regional areas that are significantly underserviced and disadvantaged [7]. It is well-established that young people living in rural and regional areas experience poorer health outcomes and greater difficulties accessing supports [8]. Further, young people and carers acknowledged that the impact of their mental illness itself may pose a barrier to young people accessing psychosocial support (e.g., social isolation, amotivation). To address these individual and systemic barriers, young people and carers identified the need for greater flexibility and responsiveness in how psychosocial support is accessed and provided in Tasmania. This includes longer duration and greater intensity of support that is aligned with and tailored to each young person’s needs, and providing assertive outreach support to engage young people creatively in their own environment. Providing flexible service delivery via telehealth or outreach, to which young people are amenable and receptive [34], may improve provision and access of supports for young people experiencing accessibility barriers.

Young people and carers reflected on negative experiences of young people’s concerns being invalidated, dismissed or judged by health professionals or family members. Young people consistently report power imbalances and not feeling heard by professionals, which often results in service disengagement and pessimism/ambivalence to access supports in future [35]. The therapeutic relationship between a young person and their support person is the most important predeterminant for positive psychosocial outcomes, particularly for those who have had adverse childhood experiences [36]. Young people require the stability and consistency of someone who is non-judgemental, empathetic, and listens. These characteristics are well-documented [37], and must be prioritised in real-world practice and recruitment of people working with young people experiencing mental illness. However, difficulties associated with finding the appropriate clinician and ensuring consistency of care are exacerbated in Tasmania due to mental health workforce shortages that are more pertinent in this state than the rest of Australia [38]. Tasmania faces significant difficulties with recruitment and retention of mental health staff, which have worsened since COVID-19, with an over-reliance of locum specialist staff and limited local candidates with the desired skills and experience [38]. Peer support/lived-experience workers were consistently suggested by both young people and carers to address some of the identified barriers, including ‘finding someone to click with’ due to similar lived experiences and identities, and to foster the youth mental health workforce. Particularly where young people lack family support networks, a peer support worker was proposed to be a consistent source of support and advocacy. Peer support has become well-established in the adult mental health sector, but less so among youth. Whilst the empirical evidence base supporting youth peer support interventions is not as robust for youth, there is a growing body of pre-post evaluation studies and qualitative research suggesting that youth peer support workers may overcome existing help-seeking barriers (e.g., stigma) and enhance the trajectory of young people with mental illness, including various domains of psychosocial wellbeing and daily functioning. [39, 40]

### Implications

This study has provided valuable insights into the perspectives of carers and young people experiencing mental illness in Tasmania. The following lived-experience recommendations informed by young people and carers may help to guide service (re)design and development, by addressing the identified barriers and enhancing the facilitators to better support young people’s psychosocial wellbeing. These findings will directly inform the co-design and development of a new psychosocial service for young people in Tasmania. As the current findings align with prior international research, such recommendations may also be adapted and implemented across youth mental health sectors internationally.

1. Development and implementation of services that can provide integrated, holistic, and flexible psychosocial support for young people in the ‘missing middle’. This may include community-based services that: (1) have broader eligibility criteria tailored for those who currently fall in the ‘missing middle’; (2) have flexible/multiple methods of access and engagement; (3) are promoted/advertised within the community to increase awareness as a step-up/step-down option to current supports to facilitate continuity of care; and (4) utilise co-location to allow young people to stay with the same service but receive different levels of care as needed.
2. Greater integration of health and social care within the youth mental health sector to better meet young people’s clinical and broader psychosocial needs (i.e., integrated ‘one-stop-shops’ or ‘youth hubs’ with multidisciplinary staff).
3. Services to employ diverse lived-experience peer support workers to work with young people experiencing mental illness.
4. Greater capacity of services to be more flexible in how they engage and support young people, including assertive outreach, telehealth, longer duration of support over 12 months, after-hours accessibility, and greater frequency/intensity of contact dependent on the young person’s needs.
5. Services to improve transparent communication with family/carers, particularly during the transition period of turning 18 years of age, by softening confidentiality laws to extend parental access to their child’s health information up to age 25 years, if their child has provided consent.
6. Facilitate or provide more access to evidence-based resources, education or practical support to carers where appropriate.

### Strengths and limitations

A key strength of this study is that young people were involved in every stage of the research. Youth experiencing mental illness are rarely included in research that concerns them, yet this should become standard practice. For this study, young people were involved in developing the aim, recruitment materials, data collection methods/questions, and supporting the analysis. This ensured that the research aligns with young people’s needs, perspectives, and experiences. Including both young people and carers in the same sample was another strength that enabled triangulation of the findings to provide a comprehensive understanding of the perspectives of two participant groups. This is particularly highlighted in the findings about fragmented communication and the differing views regarding engagement between services and families, which is crucial to informing holistic care. The sample of young people was largely representative in terms of a wide range of mental health concerns, psychosocial needs and demographic characteristics. The size of the sample, and inclusion of a broad age range of young people were also key strengths of the study that enriched the data to capture the experiences of numerous different consumers. The majority of young people had experience accessing services, which provided valuable insights into both the process of seeking support and receiving support. However, this may have biased the results as the sample represents a minority of young people with mental illness who actually access or engage in supports [11]. This bias is a limitation inherent to the purposive sampling and recruitment strategy via existing services. The study is also missing fathers’/male caregivers’ perspectives; female caregivers predominantly participate in research that concerns their child [41]. Future targeted efforts are required to capture the perspectives of male caregivers and young people who do not seek help or are unable to receive the support they need. Further, there may have been acquiescence bias present during dyad interviews due to the inherent nature of the carer-child relationship and potential power imbalance, which may have influenced the young person’s responses. Nonetheless, the findings have been triangulated and data saturation was reached, with both the dyad and individual interviews producing consistent themes. Finally, participants’ experiences are specific to the local Tasmanian context of mental health service accessibility and provision. Nonetheless, key barriers and facilitators align with international research, hence the lived-experience recommendations may have implications and generalisability for youth mental health sectors across other similar countries.

## Conclusion

The current findings highlight individual, interpersonal, and systemic barriers that young people and carers face to getting their psychosocial needs met by mental health services. Lived-experience recommendations for public mental health service design, development, policy, and practice have been provided. To better support their psychosocial functioning, young people and carers want lived-experience peer support workers to provide practical wrap-around support, and they want services that integrate health and social care, and are flexible, responsive and safe. (Re)design and development of services to address the identified barriers across the youth mental health sector is needed to empower young people, enhance their psychosocial functioning and support holistic recovery from mental illness.

## Data Availability

Data is available from the corresponding author upon reasonable request.
